# The increased antioxidant action of HDL is independent of HDL cholesterol plasma levels in triple-negative breast cancer

**DOI:** 10.3389/fonc.2023.1111094

**Published:** 2023-03-09

**Authors:** Amarilis de Lima Campos, Maria Isabela Bloise Alves Caldas Sawada, Monique Fátima de Mello Santana, Rodrigo Tallada Iborra, Sayonara Ivana Santos de Assis, Mozania Reis, Jacira Xavier de Carvalho, Luiz Henrique Gebrim, Marisa Passarelli

**Affiliations:** ^1^ Programa de Pós-Graduação em Medicina, Universidade Nove de Julho (UNINOVE), São Paulo, Brazil; ^2^ Centro de Referência da Saúde da Mulher (Hospital Pérola Byington), São Paulo, Brazil; ^3^ Hospital de Força Aérea de São Paulo, São Paulo, Brazil; ^4^ Laboratório de Lípides (LIM10), Hospital das Clínicas (HCFMUSP) da Faculdade de Medicina da Universidade de São Paulo, São Paulo, Brazil; ^5^ Physical Education Program, Universidade São Judas Tadeu, São Paulo, São Paulo, Brazil; ^6^ Unidade Básica de Saúde Dra. Ilza Weltman Hutzler, São Paulo, Brazil

**Keywords:** breast cancer, triple-negative breast cancer, HDL, LDL oxidation, antioxidant

## Abstract

**Introduction:**

The association between high-density lipoprotein cholesterol (HDLc) with the incidence and progression of breast cancer (BC) is controversial. HDL removes excess cholesterol from cells and acts as an antioxidant and anti-inflammatory. BC is a heterogeneous disease, and its molecular classification is important in the prediction of clinical and therapeutic evolution. Triple-negative breast cancer (TNBC) presents higher malignancy, lower therapeutic response, and survival rate. In the present investigation, the composition and antioxidant activity of isolated HDL was assessed in women with TNBC compared to controls.

**Methods:**

Twenty-seven women with a recent diagnosis of TNBC, without prior treatment, and 27 healthy women (control group) paired by age and body mass index (BMI) were included in the study. HDL and low-density lipoprotein (LDL) were isolated from plasma by discontinuous density gradient ultracentrifugation. Plasma lipid profile and HDL composition (total cholesterol, TC; triglycerides, TG; HDLc; phospholipids, PL) were determined by enzymatic colorimetric methods. ApoB and apo A-I were quantified by immunoturbidimetry. The antioxidant activity of HDL was determined by measuring the lag time phase for LDL oxidation and the maximal rate of conjugated dienes formation in LDL incubated with copper sulfate solution. The absorbance (234 nm) was monitored at 37°C, for 4 h, at 3 min intervals.

**Results:**

The control group was similar to the TNBC concerning menopausal status, concentrations, and ratios of plasma lipids. The composition of the HDL particle in TC, TG, PL, and apo A-I was also similar between the groups. The ability of HDL to retard LDL oxidation was 22% greater in the TNBC group as compared to the control and positively correlated with apoA-I in HDL. Moreover, the antioxidant activity of HDL was greater in the advanced stages of TNBC (stages III and IV) compared to the control group. The maximum rate of formation of conjugated dienes was similar between groups and the clinical stages of the disease.

**Discussion:**

The results highlight the role of HDL as an antioxidant defense in TNBC independently of HDLc plasma levels. The improved antioxidant activity of HDL, reflected by retardation in LDL oxidation, could contribute to limiting oxidative and inflammatory stress in advanced stages of TNBC.

## 1 Introduction

Breast cancer (BC) is the most commonly diagnosed malignant tumor in women and contributes to 69% of deaths associated with cancer. Considering its heterogeneous nature, the histological classification of BC based on the expression of receptors for estrogen, progesterone, and HER-2 helps to predict therapy and prognosis (1). Triple-negative BC (TNBC) is negative for hormone receptors and HER2 accounting for about 10-20% of all BC. It differs from other types of invasive cancer by being more prevalent in women younger than age 40, growing and spreading faster with fewer oncological therapeutic options, and tending to have a worse prognosis and survival (2).

Alterations in plasma lipid and lipoprotein profiles are contributors to BC, considering the role of lipids, particularly cholesterol, in tumor proliferation and metastasis (3, 4). Solid tumors accumulate cholesterol by increasing its synthesis and the uptake of native and modified forms of low-density lipoproteins (LDL). In TNBC, plasma lipids are reported as increased, probably helping to supply lipids to the tumor and supporting its aggressiveness. Potential lipid biomarkers (ceramides, phosphatidylcholine, lysophosphatidylcholine, and diacylglycerol) were detected in TNBC (5), reinforcing the change in lipid profile in this more aggressive tumor (2).

On the other hand, high-density lipoproteins (HDL) are considered protectors since they remove excess cholesterol from cells and minimize oxidation and inflammation (6). Nonetheless, the association of HDL cholesterol (HDLc) with BC development and progression is still controversial, largely due to the concomitant presence of metabolic comorbidities that influence the levels of HDLc in plasma, ongoing oncological therapies and intrinsic differences among hormone-sensitive tumors, and TNBC (7). Moreover, it is conceivable that, at the resemblance of the prediction of cardiovascular disease, HDLc measurement utilized as a protection metric is not sufficiently discriminating, considering the interplay of functions promoted by HDL. Markers of lipid peroxidation are enhanced in BC pointing to a role in HDL dysfunction (8). In the present investigation, the composition and the antioxidant role of HDL particles isolated from TNBC women´s plasma were measured in comparison to healthy control women.

## 2 Material and methods

Two-hundred and one women newly diagnosed with BC between 18 and 80 years old in stages I to IV of the disease, not receiving any treatment were recruited at Hospital Pérola Byington. The molecular classification of tumors was performed according to the American College of Pathologists (9, 10) in breast samples obtained by percutaneous biopsy or surgery submitted to immunohistochemistry. From this large casuistic, a convenience sample of TNBC was obtained corresponding to the 16% frequency of TNBC in population. Then, 27 TNBC women were included in the protocol. Twenty-seven health women paired by age and body mass index (BMI) were recruited at Unidade Básica de Saúde Dra. Ilza Weltman Hutzler and included as a control group. Women with diabetes mellitus, autoimmune diseases, hypothyroidism, chronic kidney disease (estimated glomerular filtration rate < 60mL/min/1.73m^2^), smokers, alcoholics, in use of antioxidants, anti-inflammatory drugs, hormone replacement therapy or contraceptives, and with a previous history of cancer, and *in situ* breast disease or actual pregnancy were not included in the study. All participants have signed an informed written consent approved by institutional Ethics Committees in accordance with the Declaration of Helsinki.

## 3 Isolation of HDL from TNBC and control women

Blood was obtained by a venous puncture after 12h fasting and the plasma was immediately isolated by refrigerated centrifugation (3,000 rpm, 4°C, 15 min). HDL (D = 1.063-1.21 g/mL) was isolated from BC and control women´s plasma by discontinuous density ultracentrifugation and immediately frozen at -80°C in a 5% saccharose solution. Plasma and HDL composition in lipids [total cholesterol (TC), triglycerides (TG), and phospholipids (PL)] was determined by enzymatic techniques. ApoB (plasma) and apo A-I (isolated HDL) were quantified by immunoturbidimetry (Randox Lab. Ltd. Crumlin, UK). HDLc plasma levels were determined after precipitation of apoB in plasma treated with dextran sulfate/magnesium chloride. Low-density lipoprotein cholesterol (LDLc) was determined by the Friedewald formula (11). Isolated HDL was extensively dialyzed against phosphate buffer saline (PBS) without EDTA immediately prior to the experiments of LDL oxidation.

### 3.1 Isolation of LDL from a healthy donor

LDL (D = 1.019-1.063 g/mL) was obtained by sequential ultracentrifugation of plasma from a unique healthy volunteer and was purified by discontinuous density ultracentrifugation. After dialysis against PBS with EDTA and sterilization, protein quantification was performed by the Lowry technique (12) and LDL kept at 4°C was utilized for experiments within 2 weeks. Samples were dialyzed against PBS without EDTA immediately before the experiments of LDL oxidation. 

### 3.2 Measurement of cooper-induced LDL oxidation in the presence of HDL isolated from TNBC and control women

The antioxidant role of HDL was accessed by determining the lag time phase for LDL oxidation and the maximal rate of conjugated dienes formation induced by copper sulfate (CuSO_4_) as previously described (13). Briefly, 40 µg of LDL protein (diluted in 500 µL of water) obtained from a single donor were incubated with 1mL of 10 μmol/L CuSO_4_ alone (final concentration) as a control incubation or in the presence of 80 µg of HDL protein from control or TNBC women at 37 °C. The absorbance at 234 nm was continuously monitored in 3 min intervals for 4 h. The time (min) of LDL resistance against oxidation (lag time phase) was calculated between the beginning of the reaction and the time interval with the extrapolated line of the propagation phase, and the maximum ratio of formation of conjugated dienes, determined by the absorbance maximum/minute (Δ absorbance/Δ min between the initiation phase and the maximal absorbance phase). The inter-assay coefficient of variation was 7.8%.

## 4 Statistical analysis

Non-parametric data were represented by the median with lower and upper quartiles The Mann-Whitney test was used for comparisons between two groups, and the Kruskal-Wallis test with the Bonferroni posttest for more than two groups. Correlation analysis was done by the Spearman test. A value of *P* < 0.05 was considered statistically significant. IBM^®^ SPSS Statistics (version 27.0), GraphPad Prisma (version 5.04) for Windows, and Microsoft^®^ Excel for Mac (version 16.52) software were used for data tabulation and analysis.

## 5 Results

Age, BMI, and menopausal status were similar between control and TNBC groups. Moreover, no differences were observed in plasma lipid profile and lipid ratios between groups (**Table 1**).

**Table 1:** Age, BMI, menopausal status, and plasma lipid profile in control and TNBC women.

	Control	TNBC
n	27	27
age (years)*	52 (43.5 – 58.5)	54 (41.0 – 61.0)
BMI (kg/m^2^)*	28 (24.4 – 29.7)	28 (24.2 – 31.6)
Menopause (%)	66.7	66.7
TC (mg/dL)	184 (142 – 201)	202 (175 – 218)
TG (mg/dL)	87 (58 – 122)	104 (96 – 144)
HDLc (mg/dL)	43 (34 – 46)	42 (37 – 48)
VLDLc (mg/dL)	17 (12 – 24)	21 (19 – 28)
LDLc (mg/dL)	117 (93 – 138)	132 (97 – 152)
non HDLc (mg/dL)	136 (104 – 160)	161 (135 – 177)
apoB (mg/dL)	98 (83 – 143)	135 (106 – 165)
TC/apoB	1.52 (1.37 – 2.06)	1.46 (1.24 – 2.02)
TG/HDLc	1.76 (1.24 – 3.12)	2.54 (2.07 – 3.46)

Plasma lipids were determined by enzymatic techniques and apoB by immunoturbidimetry. HDLc was determined after precipitation of apoB containing lipoproteins. VLDLc was calculated as TG/5, and non-HDLc, as TC-HDLc. Comparisons were done by the Mann-Whitney test; values as median and interquartile intervals. BMI, body mass index; TC, total cholesterol; TG, triglycerides; apoB, apolipoprotein B.

Women with BC were divided according to the clinical stages of the disease, as localized (stages I and II) and advanced disease (stages III and IV). There was no difference in age and BMI and plasma lipids by comparing these groups with the control group (**Table 2**).

**Table 2:** Age, BMI, and plasma lipid profile in control and TNBC women categorized according to the clinical stage of the disease.

	Control	TNBC Stages I and II	TNBC Stages III and IV
n	27	13	14
age (years)	52 (43.5 – 58.5)	54 (40 – 62)	54 (45 – 61)
BMI (kg/m^2^)	28 (24.4 – 29.7)	29 (24 – 31)	27 (24 – 31)
TC (mg/dL)	184 (142 – 201)	191 (183 – 216)	207 (172 – 226)
TG (mg/dL)	87 (58 – 122)	118 (97 – 192)	101 (92 – 132)
HDLc (mg/dL)	43 (34 – 46)	44 (32 – 63)	48 (32 – 56)
VLDLc (mg/dL)	17 (12 – 24)	24 (19 – 38)	20 (18 – 26)
LDLc (mg/dL)	117 (93 – 138)	114 (95 – 141)	138 (118 – 155)
non-HDLc (mg/dL)	136 (104 – 160)	159 (131 – 174)	164 (144 – 188)
apoB (mg/dL)	98 (83-143)	139 (110 – 171)	136 (101 – 156)
TG/HDLc	1.76 (1.24 – 3.12)	2.16 (1.94 – 2.35)	2.62 (2.33 – 2.91)
TC/apoB	1.52 (1.37 – 2.06)	1.35 (1.17 – 1.92)	1.81 (1.40 – 2.08)

Plasma lipids were determined by enzymatic techniques and apoB by immunoturbidimetry. HDLc was determined after precipitation of apoB containing lipoproteins. VLDLc was calculated as TG/5, and non-HDLc, as TC-HDLc. Comparisons were done by the Kruskal-Wallis test with the Bonferroni posttest; with values as median and interquartile intervals. BMI, body mass index; TC, total cholesterol; TG, triglycerides; apoB, apolipoprotein B.

The composition of the HDL particle in TC, TG, PL, and apo A-I was similar between the control and BC groups (**Table 3**) and among clinical stages of BC (data not shown).

**Table 3:** Composition of HDL in lipids and apoA-I in control and TNBC women.

	Control (n= 27)	TNBC (n = 27)
TC (mg/dL)	46.8 (38.0 – 57.0)	43.7 (30.2 – 55.8)
TG (mg/dL)	15.3 (11.5 – 24.3)	22.5 (14.0 – 83.7)
PL (mg/dL)	86.4 (69.7 – 102.4)	88.7 (76.5 – 115.2)
apo A-I (mg/dL)	104.4 (73.4 – 143.7)	101.2 (82.4 – 120.0)

HDL was isolated by discontinuous density ultracentrifugation and its composition in lipids and apo A-I determined by, respectively, enzymatic techniques and immunoturbidimetry. Comparisons were done by the Mann-Whitney test; values as median and interquartile intervals. TC, total cholesterol; TG, triglycerides; PL, phospholipids.

**Figure 1:**
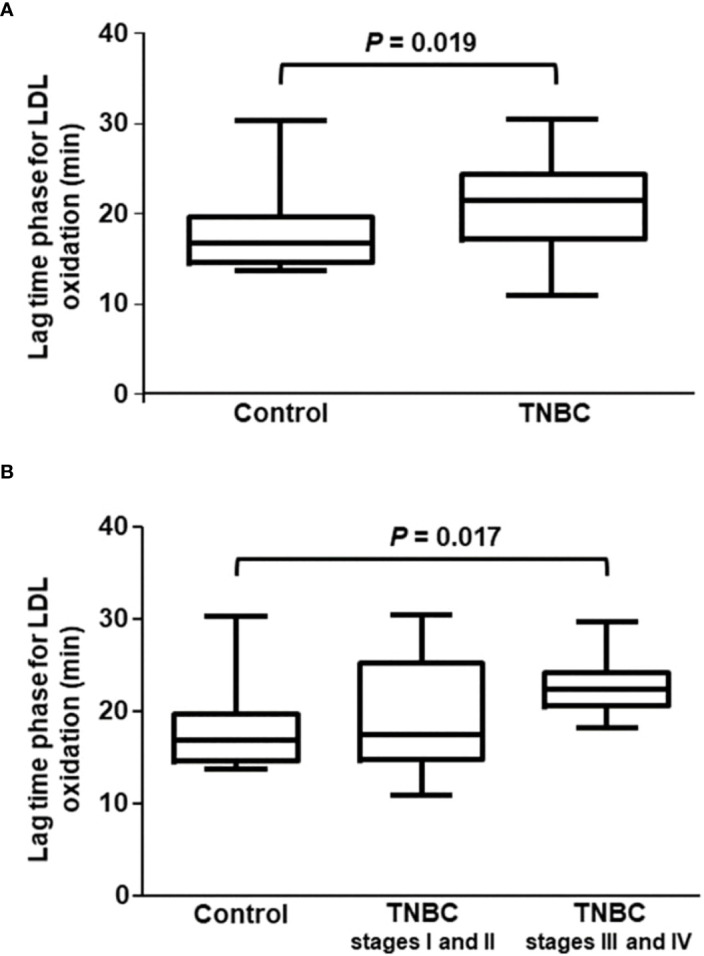
Lag time phase for CuSO_4_-induced LDL oxidation in the presence of HDL isolated from control and TNBC women. The lag time phase for LDL oxidation was determined by incubating LDL (from a unique plasma donor) with CuSO_4_ in the presence of HDL from control (n = 27) and TNBC (n = 27) women. Absorbance was monitored at 234 nm, every 3 min for 4h Comparisons were done between control and TNBC groups by the Mann-Whitney test **(A)**, and among control and the stages of BC **(B)** by the Kruskal-Wallis test with the Bonferroni posttest.

As shown in **Figure 1A**, the lag time phase for the LDL oxidation was 22% higher in the presence of HDL from TNBC women as compared to HDL from control subjects, reflecting a better antioxidant of HDL in TNBC. The lag time phase for LDL oxidation was similar between localized (stages I and II) and advanced disease (stages III and IV) but was higher in advanced disease as compared to the control group (**Figure 1B**).

**Figure 2:**
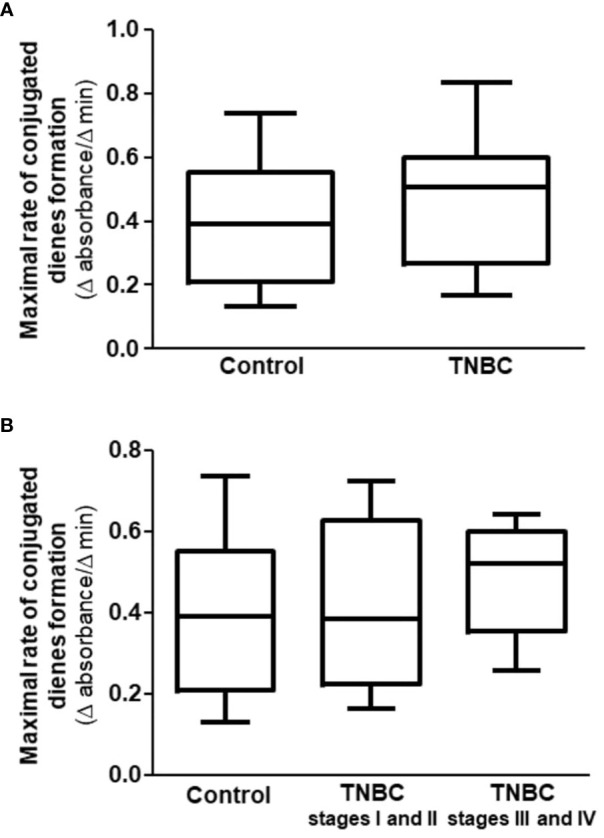
Maximal rate of conjugated dienes formation in CuSO_4_-induced LDL oxidation in the presence of HDL isolated from control and TNBC women. The maximal rate of LDL oxidation was determined by incubating LDL (from a unique plasma donor) with CuSO_4_ in the presence of HDL from control (n = 27) and TNBC (n = 27) women. Absorbance was monitored at 234 nm, every 3 min for 4 h Comparisons were done between control and TNBC groups by the Mann-Whitney test **(A)**, and among control and the stages of BC **(B)** by the Kruskal-Wallis test with the Bonferroni posttest.

The maximal rate of conjugated dienes formation was similar between control and TNBC groups and among control and BC cases according to the stage of the disease (**Figures 2A, B**).

**Figure 3:**
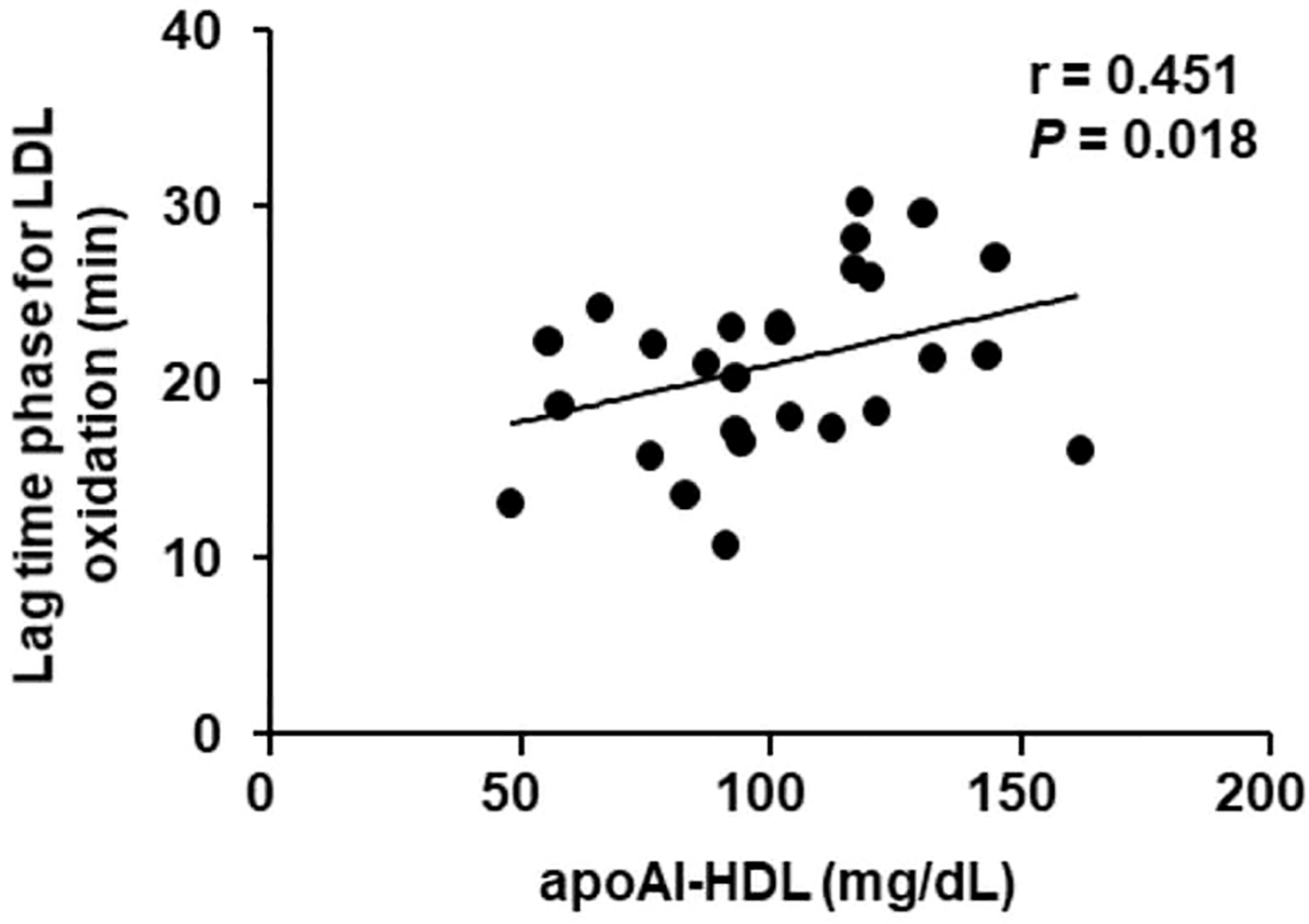
Correlation between apoA-I in HDL and the lag time phase for LDL oxidation in TNBC women. The antioxidant role of HDL isolated from TNBC women (n =27) was determined by the lag time phase for LDL oxidation induced by CuSO4. The correlation was done by the Spearman test.

The antioxidant role of HDL inferred by the lag time phase for LDL oxidation positively correlated with the concentration of apo A-I in the HDL particle (**Figure 3**).

## 6 Discussion

In the present investigation, it was demonstrated that the antioxidant capacity of the HDL particle isolated from women with TNBC is greater when compared to that of control women, particularly in the more advanced stages of the disease. This event was observed despite similar concentration of HDLc, plasma lipids, and apo A-I between groups.

Alterations in the plasma lipid and lipoprotein profile are identified as independent contributors to the risk of BC in women, regardless of menopausal status (14). Findings from several major clinical studies suggest a direct association between LDLc and BC risk and an inverse relationship between circulating HDLc and the risk of developing BC. However, these results have not been replicated in some epidemiological studies (15). Despite the lack of epidemiological evidence, the growth of benign and malignant tumor tissues has been associated with changes in plasma concentrations of lipids and lipoproteins in patients with BC (16). Several studies have found that many cancer-causing signaling pathways affect cholesterol production, meaning that cholesterol plays a role in tumor formation (4).

Although small, the casuistry of the present study showed no difference in the profile of plasma lipids, particularly HDLc, and lipid ratios between women in the control and BC groups, unlike what has been described in other studies (15, 16). Women in both groups were also similar in terms of age, BMI, and menopausal status. Even when subdivided according to the clinical stage of the disease, no differences were observed in anthropometric characteristics, menopausal status, and plasma lipids. However, components of the metabolic syndrome were not considered in this study due to the logistics of attendance and collection of samples from the participants.

HDL exerts several actions that appear to be protective against the development of many non-degenerative chronic diseases, although their association specifically with the prevention of BC is much discussed (17). This is due to the fact that the reduction of HDLc is associated with risk factors for cancer, such as menopause, diabetes mellitus, obesity, and insulin resistance (17–19).

Reduced HDLc concentrations are associated with decreased overall survival, worse prognosis and survival for TNBC (8, 20, 21), and higher incidence of BC in postmenopausal women (22). A follow-up period of 11.5 years found an inverse association between HDLc and BC risk (23), and retrospectively collected clinical data showed that HDLc reduction had a significant association with the overall risk of BC (7). Furthermore, low HDLc has been associated with more aggressive tumor characteristics (16) although HDLc concentrations lower than 50 mg/dL were modestly associated with BC risk (24). There is also evidence that the elevation of HDLc, due to genetic or drug causes, is associated with a higher risk of BC (25).

It is important to consider that the metric for inferring protection conferred by HDL - by determining the cholesterol content in the particle (HDLc) - seems to fail in predicting risk, similar to what happens for atherosclerotic macrovascular disease. This event lies in the fact that HDLc does not invariably reflect the functionality of HDL, in particular its antioxidant, anti-inflammatory, and cellular cholesterol removal activities. The controversies regarding the association between HDL and BC also reside in the face of the different studied populations and sample sizes. Disease duration, histological and molecular types, as well as influences imposed by the presence of metabolic comorbidities linked to the risk of breast tumors, oncological therapies, and changes in lifestyle, can also add bias to the analyses (26).

The HDL particle composition in CT, TG, PL, and apo A-I was similar between women in the control and BC groups and between the early and advanced stages of the disease. Thus, the greater antioxidant activity observed in HDL from the BC group (22% increase in the delay time for LDL oxidation) and, more specifically, in the advanced stages of the disease, cannot be attributed to changes in classic components of its composition. However, it is known that HDL transport a range of proteins and bioactive lipids that make up their proteomics and lipidomics. These are not easily determined by simpler laboratory techniques, but may be determinants of their activity in modulating LDL and cell membranes oxidation.

HDL anchors several enzymes in its structure, particularly paraoxonase (PON-1), which acts in the hydrolysis of lipid peroxides, minimizing LDL oxidation and the consequent supply of cholesterol and oxysterols to tumor cells (27). PON-1 concentration and activity were not determined in the present study but may contribute to the observed antioxidant response. There are studies reporting a decrease in PON-1 activity in cancer patients (28, 29). This may indicate impaired defense property against oxidative stress with potential implications for cell proliferation, promotion of gene instability, and changes in cell susceptibility to chemotherapy. There is a consistent correlation between cancer and decreased serum PON1 activity (29).

In this study, the lag time for LDL oxidation was positively correlated with apo A-I content in the HDL particle. Apo A-I is one of the components of HDL that favors the antioxidant activity of this lipoprotein (30), as well as PON-1. However, a high concentration of apo-AI was associated with a high incidence of BC (31). On the other hand, the incidence of BC was lower among women with higher apoB concentration and higher apoB/apo-AI ratio (32). These findings were surprising across all regression models. However, until recently, the association between apolipoproteins and BC was not evaluated in larger studies.

Solid tumors contain a large amount of lipids due to their increased synthesis and lipoprotein uptake (33) through scavenger receptors. In particular, the greater expression of the scavenger receptor class B type 1 (SR-B1) is linked to the greater aggressiveness of tumors and their unfavorable prognosis (34–36) while changes in its functionality, due to mutations, are related to the inhibition of tumor proliferation (37).

In a large sample of women with newly diagnosed BC, naïve to treatment, including all molecular types (n = 186), HDL composition was compared with healthy control women (n = 150). In BC, HDL was less enriched in TC, FL, and oxysterols (particularly 27-hydroxycholesterol) which may indicate less removal of cellular lipids. However, *in vitro* analysis of the intrinsic ability of HDL to remove cellular cholesterol demonstrated that cholesterol efflux from macrophages was similar between HDL isolated from BC and controls. On the other hand, in advanced stages of the disease (stages III and IV), despite the similar composition in apoA-I and lipids, HDL showed a lower ability to remove cholesterol from macrophages compared to HDL in the early clinical stages of BC (38).

Similarly to the present study, the anti-inflammatory activity of HDL was higher in BC (n = 38) compared to the control group (n = 9), regardless of the molecular type. However, in the more advanced stages of the disease (stages III and IV), the capacity of HDL to inhibit the secretion of inflammatory cytokines by macrophages was greater than in the initial stages (I and II) (unpublished data).

It is not possible, from these findings, to infer the exact contribution of HDL to tumor evolution, since it can also be modified in the tumor microenvironment. Thus, the results observed in HDL isolated from plasma may be due to reverse causation and may not necessarily reflect a causal effect on the genesis and evolution of cancer. The concept of HDL modulation by the tumor by reverse causation can unlink HDL as a direct determinant of tumor risk, being more related as a marker of tumor evolution than exactly protective or inducing its genesis. Inflammation and oxidation accompany the tumor bed and can modify HDL functionality. In this sense, inflammatory markers bind to HDL, detaching the apoA-I, which compromises its functionality (39, 40). Another limitation of the present investigation is the fact that dietary habits and physical activity were not recorded which may impact HDL generation and metabolization.

In the present study the results showed, for the first time, the role of HDL as an antioxidant defense in TNBC. This occurred independently of changes in HDL particle composition and plasma lipid profile. The greater antioxidant activity in advanced stages of TNBC, reflected by the delay in LDL oxidation even without changing the maximum ratio of conjugated dienes formation, could contribute to limiting oxidative and inflammatory stress in these tumors with worse clinical and therapeutic prognosis. By reducing LDL oxidation, HDL would reduce the supply of cholesterol and oxysterols to the tumor microenvironment, through oxidized LDL. Furthermore, it would limit the propagation of signaling pathways that result in processes of epithelial-mesenchymal transition and metastasis. Results reinforce that the determination of HDLc does not represent the best metric to infer the association of HDL with BC risk and, possibly, the evolution of the disease. Further investigation is required to better understand if the antioxidant function of HDL can contribute to the evolution of other histological types of BC. This is especially important considering the heterogeneous nature of BC, specifically related to the action of steroid hormones (estrogens and progesterone) that drives tumor evolution as well as HDL generation and metabolization.

## Data availability statement

The raw data supporting the conclusions of this article will be made available by the authors, without undue reservation.

## Ethics statement

The studies involving human participants were reviewed and approved by Universidade Nove de Julho (#3.139.460; February/2019); Centro de Referência da Saúde da Mulher (Hospital Pérola Byington; #3.225.220; March/2019); e Hospital das Clínicas da Faculdade de Medicina da Universidade de São Paulo (#3.317.909, March/2019). The patients/participants provided their written informed consent to participate in this study.

## Author contributions

Conceptualization, MP and LHG. Casuistic selection, MIBACS, MR, and JC. Methodology, ALC, MFMS, RTI, and SISA. Formal analysis, ALC, MIBACS, and MP. Investigation and data curation, ALC, MIBACS, and MP. Writing—original draft preparation, ALC and MP. Writing—review and editing, MP. Resources, MP. Project administration, MP. Funding acquisition, MP. All authors contributed to the article and approved the submitted version.
